# Therapeutic Hypothermia Following Cardiopulmonary Arrest: A Systematic Review and Meta-Analysis with Trial Sequential Analysis

**DOI:** 10.2478/jccm-2023-0015

**Published:** 2023-05-08

**Authors:** Robert Maclaren, Sterling Torian, Tyree Kiser, Scott Mueller, Paul Reynolds

**Affiliations:** 1 Department of Clinical Pharmacy, University of Colorado Skaggs School of Pharmacy and Pharmaceutical Sciences, Aurora, Colorado, USA; 2 Department of Pharmacy, TriStar Centennial Medical Center, Nashville, Tennessee, USA; 3 Department of Pharmacy, University of Colorado Hospital, Aurora, Colorado, USA; 4 Department of Pharmacy, VA Eastern Colorado Health Care System, Aurora, Colorado, USA

**Keywords:** targeted temperature, therapeutic hypothermia, cardiac arrest, cardiopulmonary arrest, neurology, arrhythmia

## Abstract

**Introduction:**

The risk-benefit profile of therapeutic hypothermia is controversial with several randomized controlled trials providing conflicting results.

**Aim of Study:**

The purpose of this systematic review and meta-analysis was to determine if therapeutic hypothermia provides beneficial neurologic outcomes relative to adverse effects.

**Material and Methods:**

MEDLINE and EMBASE databases were searched for randomized controlled trials of post-cardiac arrest patients comparing therapeutic hypothermia (^~^33 degrees Celsius) to normothermia or the standard of care (36 - 38 degrees Celsius). Data were collected using the Covidence systematic review software. Statistical analysis was performed by Review Manager software. Risk of bias, sensitivity, and heterogeneity were analyzed using the Cochran’s Collaboration tool, trial sequential analysis (TSA) software, and I2 statistic respectively.

**Results:**

A total of 1825 studies were screened and 5 studies (n=3614) were included. No significant differences existed between the hypothermia group and normothermia for favorable neurologic outcome (risk ratio [RR] 1.17, 95% confidence interval [CI] 0.97 to 1.41) or all-cause mortality (RR 0.97, 95% CI 0.89 to 1.05). When compared to normothermia, the hypothermia group had greater risk of adverse effects (RR 1.16, 95% CI 1.04 to 1.28), which was driven by the onset of arrhythmias. Subgroup analyses revealed that therapeutic hypothermia provided greater neurologic benefit in trials with a higher percentage of subjects with shockable rhythms (RR 0.73, 95% CI 0.6 to 0.88). Trial sequential analysis revealed statistical futility for therapeutic hypothermia and favorable neurologic outcome, mortality, and adverse effects.

**Conclusions:**

Therapeutic hypothermia does not provide consistent benefit in neurologic outcome or mortality in the general cardiac arrest population. Patients with shockable rhythms may show favorable neurologic outcome with therapeutic hypothermia and further investigation in this population is warranted. Any potential benefit associated with therapeutic hypothermia must be weighed against the increased risk of adverse effects, particularly the onset of arrhythmias.

## INTRODUCTION

Cardiopulmonary arrest is the result of cessation of adequate cardiac and pulmonary perfusion and is associated with a high degree of morbidity and mortality. Out-of-hospital and in-hospital arrest survival ranges from 10% to 20%, respectively [1]. If return of spontaneous circulation (ROSC) is achieved, neurologic recovery and function remain a challenge. Therapeutic hypothermia may improve neurologic outcome; however, the results of several randomized studies are conflicting suggesting the extent of benefit is inconclusive [2–6]. The result is a clinical conundrum of whether to use therapeutic hypothermia and in whom.

Therapeutic hypothermia, generally targeting mild hypothermia (32 to 34 degrees Celsius), is a strategy aimed at reducing complications following a potential anoxic injury after cardiopulmonary arrest [7]. Neuroprotective proposed mechanisms of therapeutic hypothermia include reduction of excitotoxicity, inflammation, and free radical production along with influence of neurogenesis, gliogenesis, and angiogenesis[8]. Therapeutic hypothermia has evolved over time in its time to initiation, targeted temperature, cooling strategies, duration of cooling, and temperature measurement [9]. Although therapeutic hypothermia carries the potential benefit of neuroprotective effects, this therapeutic strategy does not come without risks. Possible severe adverse effects include arrhythmias, bleeding, and infection.

The current literature regarding therapeutic hypothermia is heterogeneous, including a variety of different populations and interventions with varying results. Studies have included patients with cardiopulmonary arrest from shockable rhythms and non-shockable rhythms; protocols with varying target temperature; the utilization of several cooling methods; and differing durations of hypothermia. The 2017 Neurocritical Care Society guidelines for therapeutic hypothermia define targeted temperature management (TTM) as encompassing therapeutic hypothermia, controlled normothermia, and treatment of fever [7]. Recommendations from this group consist of cooling for at least 24 hours in out-of-hospital cardiac arrest, but do not make a recommendation for the goal targeted temperature (e.g. mild hypothermia or targeted normothermia). Conversely, both the 2020 American Heart Association and 2021 European Resuscitation Council give a recommendation for targeted temperature management with a goal temperature of 32 to 36 degrees Celsius for comatose post-cardiac arrest patients[10,11]. However, since the publication of these guidelines, recent randomized controlled trials have evaluated therapeutic hypothermia and resulted in conflicting results [4,6].

The goal of our systematic review and meta-analysis was to analyze the highest quality available literature and determine if mild hypothermia provides benefit in neurologic outcomes or mortality, evaluate if further randomized controlled trials are futile to conduct, and to assess whether this intervention results in increased adverse events. Additionally, we aim to determine if any subgroups in particular benefit from therapeutic hypothermia.

## MATERIALS AND METHODS

This systematic review and meta-analysis followed Preferred Reporting Items for Systematic Reviews and Meta-Analyses Guidelines and the Cochrane Handbook for Systematic Reviews [12–15]. The study protocol was submitted to PROSPERO, the international prospective register of systematic reviews, and was approved prior to the completion of the literature search (Study ID: CRD42021278407).

### Eligibility Criteria

The studies we included encompassed randomized controlled trials of patients following cardiopulmonary arrest, which compared mild therapeutic hypothermia (temperatures consisting of 32 to 34 degrees Celsius) to normothermia or the standard of care (36 to 38 degrees Celsius). Exclusion criteria consisted of studies that had no comparator group, retrospective analyses, studies examining therapeutic mild hypothermia for indications other than post-cardiac arrest, and studies involving pediatric patients (age < 18 years).

### Primary and Secondary Outcomes

The primary outcome of this study was to evaluate the degree of favorable neurologic outcome following TTM at study follow up. A favorable neurologic outcome was defined as a modified Rankin Scale (mRS) score of 0 to 2, a Cerebral Performance Category (CPC) score of 1 to 2, or the closest relevant score on an any alternate system. Secondary outcomes included all-cause mortality at longest study reported follow-up period and serious adverse events including arrhythmias, major bleeding, documented infection, and seizure defined by each respective study. A subgroup analysis was planned *a priori* and aimed to determine factors that may be associated with a favorable neurologic outcome. These subgroups included the following: shockable arrhythmia, bystander-performed cardiopulmonary resuscitation (CPR), and duration of cooling. As individual patient data were not available for the subgroup analyses, the median time or frequency across all included studies was calculated, and the outcome was assessed dichotomously as either greater than the median time or frequency or less than or equal to the median time or frequency for the aforementioned subgroups.

### Search Strategy

Both MEDLINE and EMBASE were searched using a comprehensive population, intervention, comparator, and outcome (PICO) question and MeSH terms. We also searched references listed in retrieved trials, guidelines, systematic reviews, and other meta-analysis to identify potential studies to include. Two investigators (SCT and PMR) independently screened the abstract, title, or both to determine which studies had possibility for inclusion. Covidence software (Melbourne, Australia 2022) was utilized to upload search results and conduct bias risk assessments. Potential articles for inclusion were further examined by each investigator (SCT and PMR) to review for inclusion and exclusion criteria in the analysis. Discrepancies between reviewers were discussed further, and if unable to be resolved between the two investigators, a third reviewer (RM) was involved to determine if the study met inclusion or exclusion criteria.

### Data Extraction

Information was manually extracted into a standardized data extraction form. Demographics, such as age and sex, were recorded, in addition to other information, including the number of patients with a shockable rhythm, a bystander-performed CPR, and an out-of-hospital cardiac arrest. TTM information, including time to ROSC, time from ROSC to TTM initiation, time to goal temperature, cooling and rewarming methods, and duration of cooling were recorded. Outcomes, including neurologic outcomes and mortality, were collected as reported in each study and recorded within the extraction form. Adverse effects were subject to the definitions used per each trial protocol and were collected if they were reported in a per-participant methodology. If outcomes were unavailable in the published data, attempts were made to contact corresponding authors for the included studies.

### Statistical Analysis

The Cochran’s Collaboration tool was utilized to assess for risk of bias in a blinded manner. This tool assesses bias of each study according to the following: random sequence generation, allocation concealment, blinding, incomplete outcome data, selective reporting, and other potential sources of bias. To assess the risk of publication and reporting bias, an Egger’s test was performed to evaluate funnel plot asymmetry.

Review Manager 5.4 (London, England 2020) was used for statistical analysis, in which all outcomes were dichotomous in nature. The Cochran-Mantel-Haenszel Method was used to calculate risk ratios for outcomes using a significance level of 0.05. Due to differing methodology and study interventions, a random-effects model was pursued in analyzing outcomes. Heterogeneity between trials were assessed by I^2^ statistic. Trial sequential analysis (TSA) software was used to test model robustness using an O’Brien-Fleming alpha-spending approach. The TSA takes into account heterogeneity of studies in addition to estimation of information size to better control for type 1 and type 2 errors.

## RESULTS

### Study Participants

A total of 1825 studies were identified via search on MEDLINE and EMBASE with five being included in the analyses (Figure S1) [2–6]. The main reasons for exclusion were post-hoc analyses of an included study, wrong study design, and wrong comparator. Of the included studies, all were multicenter. A total of 3611 subjects were included in the primary analysis of neurologic outcome. The majority of these studies included subjects with an initial shockable rhythm (2465 subjects) and out-of-hospital cardiac arrest (3475 subjects). Only one study included exclusively non-shockable rhythms (Table 1) [6].

**Table 1.** Characteristics of Included Studies

Study	Mild Hypothermia	Standard of Care	Initial Shockable Rhythm	Out-of-Hospital Arrest	Median Time to ROSC	Bystander CPR	Duration of Cooling	Surface Cooling
Bernard. HACA. 2002.	32 to 34	Normothermia	96%	87%	22	46%	24	100%
Bernard. OHCA. 2002.	33	Normothermia	100%	100%	26	58%	18	100%
Nielsen. TTM. 2013.	33	36	80%	100%	25	73%	28	76%
Lascarrou. HY PERION. 2019.	33	37	0%	73%	NR	70%	24	85%
Dankiewicz. TTM2. 2021.	33	37.5	74%	100%	25	80%	28	70%

NR, Not reported

### Interventions

All of the included studies compared some degree of mild hypothermia to normothermia or the reference standard of care. The target hypothermia for majority of studies [2,4–6] was 33 degrees Celsius, except for one trial[3] in which the goal was 32 to 33 degrees Celsius. The control groups varied in their target temperature or did not implement temperature management (Table 1).

### Risk of Bias Assessment

No trial met criteria for low risk of bias due to the infeasibility to adequately blind participants and treating physicians. Risk of bias assessments are displayed in the Supplementary Appendix (Figure S2). Furthermore, the Egger’s test was negative for publication bias (Figure S3).

### Outcomes

#### Favorable Neurologic Outcome

**Fig. 1.**
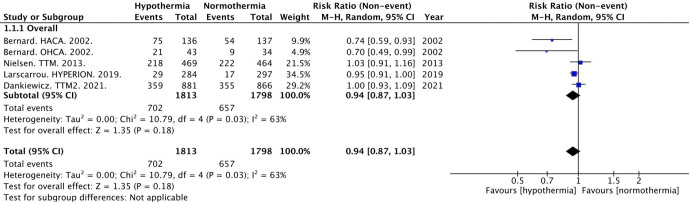
**Forest Plot for Favorable Neurologic Outcome.** Abbreviations: M-H, Mantel-Haenszel random effects; CI, confidence interval.

The primary outcome of neurologic outcome was available for 3611 subjects. Of the included trials, four trials used the Cerebral Performance Category (CPC) or similar scale for determining neurologic outcomes [2,3,5,6]. The remaining trial utilized the modified Rankin Scale (mRS)[4] A follow up duration of six months was used for the favorable outcome in three studies[2,4,5]. The remaining two studies used hospital discharge [3] and 90 days [6] for their outcome follow up period. Of the included studies, 38.7% (702/1813) and 36.5% (657/1798) of the participants had a favorable outcome in the hypothermia and normothermia groups, respectively (Table S1). The random effects meta-analysis revealed no significant difference between the two groups in favorable neurologic outcome (risk ratio [RR] 0.94, 95% confidence interval [CI] 0.87 to 1.03) with a heterogeneity of 63% (Figure 1).

#### All-Cause Mortality

**Fig. 2.**
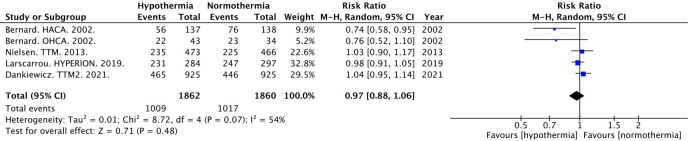
Forest plot for All Cause Mortality at Longest Follow-Up Interval. Abbreviations: M-H, Mantel-Haenszel random effects; CI, confidence interval.

A total of 3613 subjects from the five trials had follow up data available for all-cause mortality. Longest follow up for all-cause mortality mirrored that of the longest follow up for neurologic outcomes, with majority of studies reporting the outcome at six months. Overall, mortality was 54.2% (1009/1862) and 54.7% (1017/1860) in the hypothermia and normothermia groups, respectively (Table S1). There was no significant difference in all-cause mortality between groups (RR 0.97, 95% CI 0.88 to 1.06) with a heterogeneity of 54% (Figure 2).

#### Adverse Effects

**Fig. 3.**
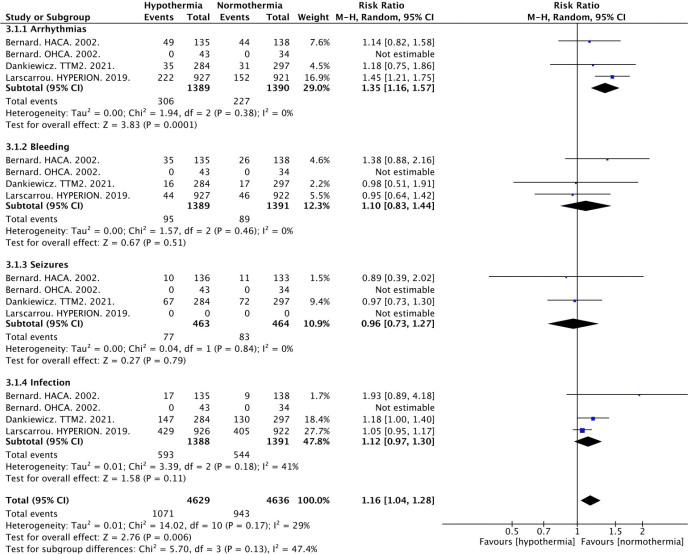
**Adverse Events.** Abbreviations: M-H, Mantel-Haenszel random effects; CI, confidence interval.

Definitions for each adverse effect were extracted from each study (Table S2). All except one trial [5] presented data in a per-participant methodology, leaving approximately 2780 subjects available for the analysis of arrhythmias, bleeding, and infection. Seizures were reported at a per-participant level for 927 subjects. Our analysis revealed the hypothermia group had a statistically significant increase in the risk of adverse effects (23.1% vs. 20.3%, RR 1.16, 95% CI 1.04 to 1.28) with a heterogeneity of 47.4% (Figure 3). When adverse effects were assessed by sub-type, arrhythmias occurred in significantly more patients in the hypothermia group compared to the normothermia group (22% vs. 16.3%; RR 1.35; 95% CI 1.16 to 1.57). Other adverse effects including bleeding, seizures, and infections were not different between groups (Figure 3 & Table 2).

**Table 2.** Absolute Percentages of Adverse Effects at the Participant Level^a^

Adverse Effect	Hypothermia	Normothermia	Absolute Difference	Fragility Index^b^
Arrhythmias	306/1389(22%)	227/1390 (16.3%)	5.7%	38
Bleeding	95/1389 (6.8%)	89/1391 (6.4%)	0.4%	0
Seizures	77/463 (16.6%)	83/464 (17.9%)	−1.3%	0
Infection	593/1388 (42.7%)	544/1391 (39.1%)	3.6%	0

a Expressed as the total number of events / total number of participants (%).

b Fragility index is described as the number of patients needed to cross over in order to generate a non-significant outcome and is a measure of robustness of a study finding

#### Subgroup Analyses

When comparing the available data of the five trials, three specified subgroups *a priori* had meaningful data to perform subgroup analyses. Subgroups were divided into the following: initial shockable rhythm (studies with > 80% vs. < 80% initial shockable rhythm), bystander CPR (studies with > 70% vs. < 70% bystander CPR), and duration of cooling (studies with > 24 hours vs. ≤ 24 hours of cooling). Of the subgroup analyses performed, studies with a higher percentage of patients with initial shockable rhythm (Figure 4) were more likely to have a favorable neurologic outcome (53.6% vs. 36.8%, RR 0.73, 95% CI 0.6 to 0.88). The remaining subgroup analyses on neurologic outcome revealed no significant differences between groups and are reported in the supplementary appendices (Figures S4 & S5). Additionally, with the signal of favorable neurologic outcome in trials with larger percentages of patients with shockable rhythms, we conducted a subgroup analysis for the outcome of mortality. Four trials, representing 1685 subjects, reported individual patient data for this analysis [2,3,5,6]. Mortality was not different in this subgroup analysis (RR 0.92, 95% CI 0.8 to 1.05) (Figure S6).

#### Trial Sequential Analysis

TSA revealed the z-curve for outcomes of favorable neurologic outcome and mortality crossed the lines of futility, signifying randomized controlled trials with these populations would not demonstrate a benefit and would be considered futile (Figures S7-S8). Furthermore, the z-curve for the outcome of adverse effects crossed the line of harm, suggesting that if the systematic review was a randomized trial that it may have been stopped early by an independent data safely monitoring committee due to risk of harm (Figure S9).

## DISCUSSION

The results of this systematic review suggest there is no benefit of mild therapeutic hypothermia with respect to favorable neurologic outcome and mortality. Our subgroup analysis also demonstrated an increased risk of adverse effects with the hypothermia group in comparison to the group receiving normothermia, particularly driven by incidence of arrhythmias. Despite the theoretical coagulopathic and immunosuppressant effects, we did not find an association between hypothermia and bleeding or infection. To our knowledge, our findings present the most comprehensive analysis of adverse effects from therapeutic hypothermia following cardiac arrest. Subsequently, our meta-analysis revealed new findings for futility of further randomized controlled trials across all of the aforementioned outcomes through TSA.

### Favorable Neurologic Outcome

Favorable neurologic recovery is arguably one of the most important outcomes with regard to post-cardiopulmonary arrest. Clinically, favorable neurologic outcome is defined by the mRS or CPC scores, which describe the functionality of a patient after suffering a neurologic injury [16,17]. Similar to previous meta-analyses, our meta-analysis revealed no overall neurologic benefit of therapeutic hypothermia when compared to normothermia [18–23]. TSA of favorable neurologic outcome demonstrated the futility of therapeutic hypothermia compared to normothermia, suggesting further randomized controlled studies should likely not be conducted with this outcome. The finding of futility conflicts with a previous meta-analysis, which is likely due to the inclusion of a larger number of lower quality studies in the aforementioned meta-analysis [22]. Our meta-analysis aimed to include the highest quality available literature, of which the referenced studies from their meta-analyses that did not meet our inclusion criteria [24–26]. Additionally, other meta-analyses have evaluated the benefit of therapeutic hypothermia with respect to subgroups of actively or passively controlled normothermia, pre-hospital cooling, and according to the type of rhythm[18,20,22]. Due to our strict inclusion criteria, we included fewer studies in our analysis, resulting in less overall data available for sub-group analyses. In our analysis, the only subgroup revealing a favorable neurologic outcome with therapeutic hypothermia consisted of studies with higher degree (> 80%) of patients with shockable rhythms [2,3].

Limitations of this outcome in our meta-analysis are important to note. One of the included trials in the analysis utilized their own neurologic outcome scale, which was not validated in previous studies [3]. However, the scale was similar to the CPC scale and was categorized accordingly for our analysis. Additionally, due to the strict nature of our inclusion and exclusion criteria and focus on the highest quality available literature, only a handful of trials met final criteria for inclusion. Many studies analyzing therapeutic hypothermia were excluded due to methodology (e.g. retrospective studies or post-hoc analyses) and comparators or interventions (e.g. assessing different durations of hypothermia, lower temperature hypothermia to standard hypothermia, and pre-hospital cooling), and, therefore, could not be considered for our primary outcome. The demonstrated benefit for favorable neurologic outcome in the shockable rhythm subgroup analysis could be confounded by the lack of actively controlled normothermia in the two included trials in the subgroup. Additionally, due to the available published data, we were only able to perform subgroup analyses by overall trial averages, limiting our ability to analyze these subgroups at a patient level. Another limitation to be considered is that trials included in this analysis were conducted over a large time span of which post-cardiac arrest management has greatly improved. However, we included TSA and alpha spending as part of our analysis in order to adjust for this change in standard of care over time.

#### All-Cause Mortality

Whether therapeutic hypothermia improves survival is controversial, especially if neurologic outcome is not favorable. Our results are similar to previous meta-analyses that demonstrated no survival benefit with hypothermia [18–23,27]. In parallel with the analysis by Sanfilippo and colleagues, our TSA confirmed the futility of further randomized controlled trials in respect to the mortality outcome [22]. All of these findings combined suggest therapeutic hypothermia in the general cardiac arrest population is likely not to provide a survival benefit. However, the question regarding benefit in select populations remains, and our subgroup analysis of individual patient level data showed a non-significant trend towards improved survival. Even though neurologic outcome showed benefit with hypothermia in the shockable rhythm subgroup analysis, mortality was not different leading to discrepancies of which outcome is most important.

This outcome was also associated with limitations necessary to note. Each of these trials reported mortality at variable follow up durations, which ranged from hospital discharge to six months. Although unlikely to confound our findings with the use of a random effects model, it is important to note these outcomes were measured at differing time points. Additionally, another important limitation was for our subgroup analysis on shockable rhythms. The recent randomized control trial by Dankiewicz and colleagues did not report individual patient data in regards to mortality and shockable rhythms [4]. Although, this would have been unlikely to influence our findings as the reported confidence interval for this subgroup in the trial demonstrated no difference.

#### Adverse Effects

Adverse effects are important when considering any therapeutic strategy to determine the risk to benefit ratio for the patient. Therapeutic hypothermia is not a benign therapy and has been associated with various adverse effects. Few of the previous meta-analyses have reported on adverse effect findings; however, of those referenced, patients randomized to therapeutic hypothermia were at an increased risk for arrhythmias [18,19,21,22]. Our study revealed consistent results in comparison to previous meta-analyses, with therapeutic hypothermia patients having a 35% increased relative risk of experiencing an arrhythmia. In contrast to prior meta-analyses, our study provided a thorough picture of the potential adverse effects of therapeutic hypothermia through examining a comprehensive list of adverse effects and their respective incidences, including bleeding (6.8%), infections (42.7%), and seizures (16.6%). Although we found no independent differences in these outcomes compared to the normothermia group, when compiled together, patients in the therapeutic hypothermia group remained at a 16% increased relative risk for adverse effects. To our knowledge, our study is the first to perform a TSA on adverse effect outcomes, which revealed futility of therapeutic hypothermia.

Important limitations exists in regards to adverse effect reporting and our analysis. First, each trial utilized their own method for reporting, with some trials using an independent data safety monitoring committee, while others were subjected to physician reporting. Due to each trial using their own definitions for adverse effects, we were unable to delineate the predominant arrhythmia reported. Similarly, the standard of care for adverse effect reporting has changed over time and become more objective according to the requirements of independent data safety monitoring committees; however, our analysis with TSA and alpha spending is aimed to adjust for this change, as mentioned previously. Additionally, one trial reported adverse effects over a total of reported events, rather than per participant, hindering our ability to include those adverse effects in our initial subgroup analysis [5]. Administration of prophylactic lidocaine therapy was performed in one of the trials and could have led to an under-reporting of arrhythmias [3]. Additionally, pre-existing structural heart disease and post-cardiac arrest myocardial dysfunction predispose patients to arrhythmias and may have contributed to the reported adverse effects in both groups [28]. Other adverse effects of hypothermia are also important to note and were not consistently reported in these studies, which include metabolic disorders and electrolyte abnormalities.

#### Future Directions

In regards to future directions of randomized controlled trials, TSA revealed futility of further trials in the comparison of hypothermia to normothermia with the outcomes of mortality, neurologic outcomes, and adverse effects. A potential direction for further analyses is prevention of pyrexia in post-cardiac arrest management and re-focusing TTM efforts on patients with shockable rhythms. Pyrexia increases metabolic demand and can lead to consequential cerebral inflammation, increased cerebral blood flow and subsequent elevated intracranial pressure, and neurotransmitter excitotoxicity [29,30]. Following cardiac arrest, occurrence of pyrexia has been associated with unfavorable outcomes [31–34]. Earlier studies with significant survival and neurologic benefit of hypothermia did not actively prevent pyrexia in the control group [2,3]. With no apparent survival or neurologic benefits of therapeutic hypothermia and the risk of adverse effects, perhaps future trial considerations should consist of prevention of pyrexia from a pharmacologic and physiologic perspective in patients with targeted normothermia of less than 38 degrees Celsius.

## CONCLUSIONS

Therapeutic hypothermia does not appear to result in a favorable neurologic outcome or survival benefit in comparison to normothermia in the general population presenting with cardiac arrest. Our subgroup analyses showed a signal towards favorable neurologic outcome with therapeutic hypothermia in shockable rhythm. Additionally, an increased risk of adverse effects, particularly driven by arrhythmias, was observed in the therapeutic hypothermia group. Our results are consistent with previous meta-analyses and present new findings of futility for further randomized controlled trials assessing therapeutic hypothermia compared to normothermia with the outcomes of favorable neurologic outcome, mortality, and adverse effects.
